# miR-31-5p Is a LIPUS-Mechanosensitive MicroRNA that Targets HIF-1α Signaling and Cytoskeletal Proteins

**DOI:** 10.3390/ijms20071569

**Published:** 2019-03-28

**Authors:** Viviana Costa, Valeria Carina, Alice Conigliaro, Lavinia Raimondi, Angela De Luca, Daniele Bellavia, Francesca Salamanna, Stefania Setti, Riccardo Alessandro, Milena Fini, Gianluca Giavaresi

**Affiliations:** 1 IRCCS Istituto Ortopedico Rizzoli, 40136 Bologna, Italy; valeria.carina@ior.it (V.C.); lavinia.raimondi@ior.it (L.R.); angela.deluca@ior.it (A.D.L.); daniele.bellavia@ior.it (D.B.); 2 Department of BioMedicine, Neuroscience and Advanced Diagnostics (Bi.N.D), University of Palermo, 90100 Palermo, Italy; alice.conigliaro@unipa.it (A.C.); riccardo.alessandro@unipa.it (R.A.); 3 IRCCS Istituto Ortopedico Rizzoli, Laboratory of Preclinical and Surgical Studies, 40136 Bologna, Italy; francesca.salamanna@ior.it (F.S.); milena.fini@ior.it (M.F.); gianluca.giavaresi@ior.it (G.G.); 4 IGEA SpA, 41012 Carpi (Modena), Italy; s.setti@igeamedical.com; 5 Institute of Biomedicine and Molecular Immunology (IBIM), National Research Council, 90100 Palermo, Italy

**Keywords:** hypoxia, mesenchymal stem cells, microRNAs, Rho family protein, regenerative medicine

## Abstract

The roles of low-intensity pulsed ultrasound (LIPUS) and microRNAs (miRNAs) on hMSCs commitments have already been investigated; however, the effects of the application of their co-treatments in an in vitro cell model are still unknown. Our previous studies demonstrated that (i) LIPUS modulated hMSCs cytoskeletal organization and (ii) miRNA-675-5p have a role in HIF-1α signaling modulation during hMSCs osteoblast commitment. We investigated for the first time the role of LIPUS as promoter tool for miRNA expression. Thanks to bioinformatic analysis, we identified miR-31-5p as a LIPUS-induced miRNA and investigated its role through in vitro studies of gain and loss of function. Results highlighted that LIPUS stimulation induced a hypoxia adaptive cell response, which determines a reorganization of cell membrane and cytoskeleton proteins. MiR-31-5p gain and loss of function studies, demonstrated as miR-31-5p overexpression, were able to induce hypoxic and cytoskeletal responses. Moreover, the co-treatments LIPUS and miR-31-5p inhibitor abolished the hypoxic responses including angiogenesis and the expression of Rho family proteins. MiR-31-5p was identified as a LIPUS-mechanosensitive miRNAs and may be considered a new therapeutic option to promote or abolish hypoxic response and cytoskeletal organization on hMSCs during the bone regeneration process.

## 1. Introduction

Low-intensity pulsed ultrasounds (LIPUS) are mechanical waves able to transmit energy through tissues, transforming mechanical energy into biological effects by causing microstreaming and stable cavitation that modify extra cellular matrix (ECM) proteins and cell permeability [1]. Various in vitro and in vivo studies have shown that LIPUS improves bone tissue mesenchymal stem cell (MSCs) proliferation, osteoblast differentiation, and cytoskeletal modifications [2,3,4].

Recently, it has been demonstrated in in vitro studies that LIPUS improves the balance between stemness and osteoblast differentiation of hMSCs by modulating different proteins such as transforming protein RhoA [3,4,5,6,7]. RhoA is a protein of the Rho Family that acts as a molecular switch responding to cell surface receptors for various cytokines, growth factors, adhesion molecules, and G-protein-coupled receptors [8,9]. It was observed that RhoA signaling cascade plays an essential role in the migration ability of hMSCs [8], particularly in hypoxic conditions in which upregulation of hypoxia inducible factor-1 (HIF-1α) and activated GTP-bound RhoA were found, highlighting a strong link between them in hMSC [10,11] and in tumor cells [12,13,14].

The role of miRNAs in the osteoblast differentiation process or stem cells niche maintenance in correlation with HIF-1α signaling activation has also been investigated [15,16]. MiR-675-5p was hypothesized to be a trigger of complex molecular mechanisms that could promote osteoblast differentiation of hMSCs during hypoxia bone formation. This would occur through a dual strategy: Increasing HIF-1α response and activating Wnt/β-catenin signaling [15].

Since miRNAs are known to have a pleiotropic role, there might be an involvement of miRNAs behind the correlation between cytoskeletal reorganization or RhoA modulation and HIF-1α signaling activation [17]. Thanks to the use of bioinformatics analysis, many genes regulated by LIPUS, such as hypoxia-inducible factor 1-α inhibitor (HIF1AN), RhoA, and Ras-related C3 botulinum toxin substrate 1 (RAC1), were evaluated as predicted or validated targets of hsa-miR-31-5p (miR-31-5p) [3,18,19]. MiR-31 has been identified to perform as a regulator of the osteogenesis of hMSCs [20,21,22] and its expression was progressively decreased in human bone marrow derived stem cells undergoing osteogenesis and in osteosarcoma cell lines, highlighting a potential role in osteogenic differentiation [21,23,24,25].

Recently, evidence has emerged on mechano-sensitive miRNAs, but their response to different mechanical stimuli and their effects on osteoblast differentiation merit further investigation. Nevertheless, recent studies have highlighted that miR-33a-5p might be classified as a mechano-sensitive miRNA [26]. In this regard, in accordance with miR-31 data reported in the literature, we hypothesized that, as suggested for miR33a-5p, LIPUS stimulation induces miR-31 expression at an early culture time point, allowing an increase in HIF1A expression, which is an inductor of MSCs osteoblast differentiation [3,15,26]. The present study aimed at demonstrating for the first time that miR-31-5p expression may be modulated by LIPUS stimulation, acting on cytoskeletal organization and HIF-1α signaling, which is the pivotal trigger of osteoblast commitments.

## 2. Results

### 2.1. LIPUS Stimulation Induces the Modulation of Cytoskeletal Proteins

**Figure 1:**
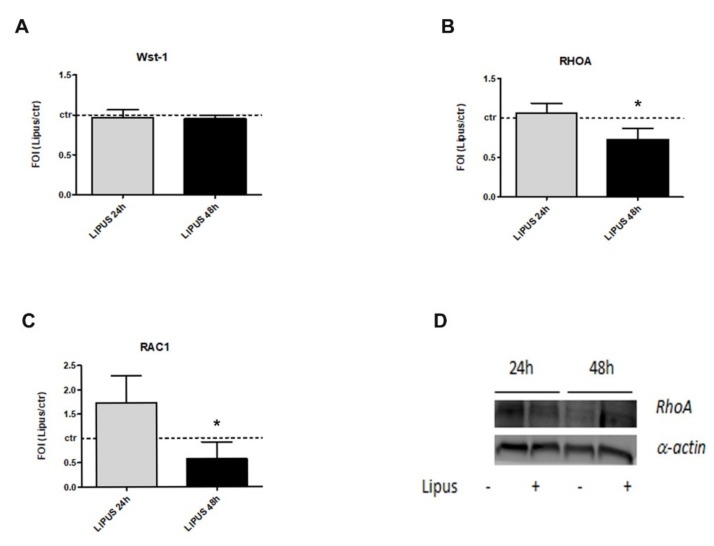
Effects of low-intensity pulsed ultrasound (LIPUS) stimulation on cytoskeletal proteins modulation in terms of cell viability (**A**), expression of RhoA (**B**) and Ras-related C3 botulinum toxin substrate 1 (RAC1) (**C**) genes, and synthesis of RhoA protein (**D**). Human MSCs stimulated with LIPUS for 24 h and 48 h showed no differences in cell viability by WST1 assay compared to LIPUS and the untreated group. Quantitative RT-PCR data are expressed as fold of change (FOI) in gene expression (2^−ΔΔ*C*t^) and occurred in LIPUS respect to untreated groups. RhoA and α-actin protein modulations were evaluated by western blot analysis (**D**). Student’s *t* test: *, *p* < 0.05 between experimental time.

WST-1 assay showed no significant modulation of LIPUS on hMSCs viability compared to the untreated control group at the early phase of treatment (24 and 48 h) (Figure 1A). LIPUS stimulation induced the down-regulation of RhoA (mRNA) compared to untreated cultures at 48 h (*p* = 0.039), followed by a significant decrease in protein during the experimental times, as showed by western blot (Figure 1B–D and Figure S1). In addition, RAC1 (mRNA) was significantly downregulated by LIPUS stimulation, supporting its role as modulator of cytoskeletal proteins (*p* = 0.023, Figure 1C).

### 2.2. LIPUS Stimulation Induces the Expression of HIF-1α

**Figure 2:**
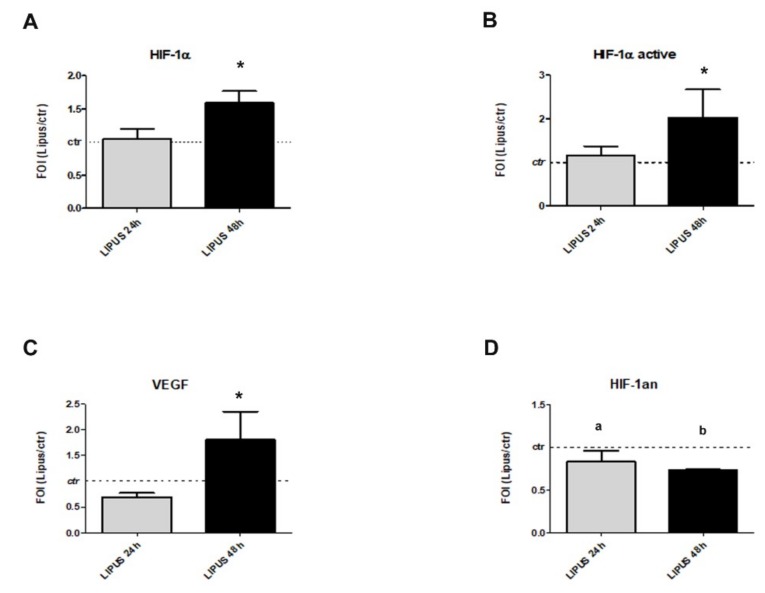
Effects of LIPUS stimulation on hypoxia inducible factor-1 (HIF-1α) expression were evaluated by gene expression of HIF-1α (**A**), VEGF (**C**), hypoxia-inducible factor 1-alpha inhibitor (HIF1AN) (**D**) and proteins analysis of HIF-1α active (**B**). Quantitative RT-PCR data are expressed as fold of change (FOI) in gene expression (2^−ΔΔ*C*t^) and occurred in LIPUS compared to untreated groups. ELISA data are expressed as FOI between hMSCs and were stimulated with LIPUS for 24 and 48 h respect to untreated group. Student’s *t* test: *, *p* < 0.05 between experimental time; a: *p* < 0.05; b: *p* < 0.0005 between LIPUS and untreated groups at each experimental time.

To investigate the preliminary role of LIPUS stimulation on HIF-1α signaling, a qRT-PCR expression profile was carried out. Firstly, HIF-1α expression was up-regulated in LIPUS groups over time points (*p* = 0.011, Figure 2A), as well as in terms of nuclear translocation of HIF-1α protein (*p* = 0.045, Figure 2B). The transcriptional activity of HIF-1α after LIPUS stimulation was confirmed by the increase of its target VEGF, the pro-angiogenic growth factor that showed an increase of its gene expression (*p* = 0.016, Figure 2C). Whereas a down-regulation of HIF1AN (mRNA) was observed after LIPUS stimulation at 24 h (*p* = 0.017) and 48 h (*p* < 0.0005) in comparison to untreated cultures (Figure 2D).

### 2.3. LIPUS Stimulation Promotes MiR-31-5p Expression

**Figure 3:**
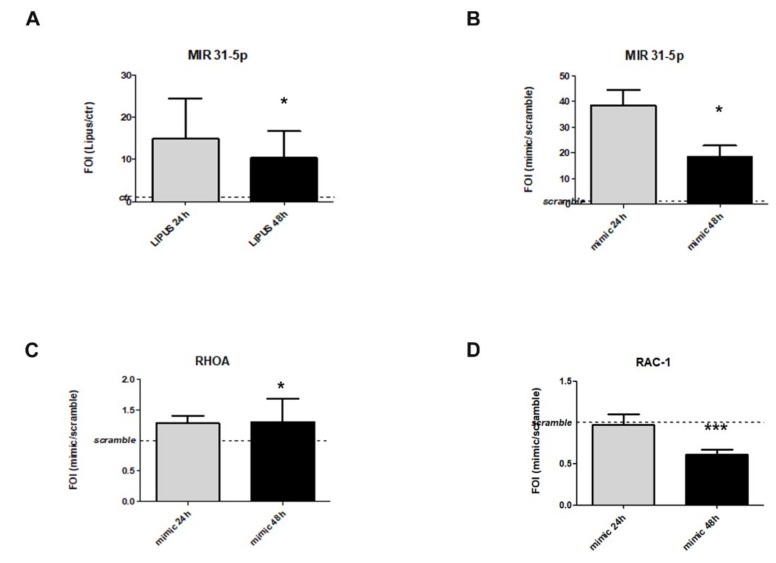
Study of miR-31-5p as a possible target of LIPUS stimulation. Analysis of miR-31-5p expression levels after LIPUS stimulation and after 24 h of miR-31-5p mimic or negative scramble transfection (**A**,**B**). Analysis of RhoA (**C**) and RAC1 (**D**) expression levels after miRNAs transfection. Quantitative RT-PCR data are expressed as fold of change (FOI) in gene expression (2^−ΔΔ*C*t^) and occurred in mimic respect to scramble groups. Student’s *t* test: *, *p* < 0.05: ***, *p* < 0.005 between experimental time.

In order to investigate the molecular mechanism driving LIPUS effects on HIF1AN, RhoA, and RAC1 gene expression, the possible involvement of miR-31-5p was taken into account. After LIPUS stimulation, hMSCs were able to express miR-31-5p compared to the untreated group (*p* < 0.05) (Figure 3A); while no changes were observed for miR-31-3p (data not shown). By MiRbase database analysis, we identified HIF1AN, RhoA, and RAC1 as predicted targets for miR-31-5p [27].

To support the role of miR-31-5p as mediator of hMSCs LIPUS effects, we transfected hMSCs cells with 15 pg/mL of miR-31-5p mimic, and a significant increase of miR-31-5p, which was observed in comparison to untreated cells (*p* = 0.019, Figure 3B). As indicated in Figure 3C,D, miRNA transfection induced a modulation of RhoA (*p* = 0.019) and RAC1 (*p* < 0.0005) mRNAs expression.

**Figure 4:**
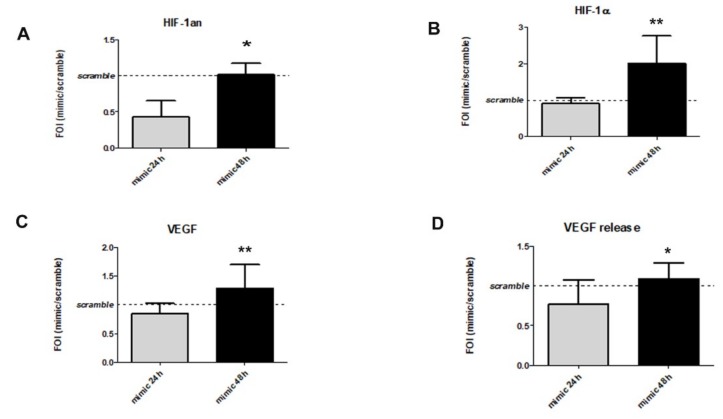
Study of HIF-1α signaling as a possible target of miR-31-5p overexpression, by analysis of HIF1AN (**A**), HIF-1α (**B**), and VEGF (**C**) gene expression and VEGF protein release (**D**). Quantitative RT-PCR data are expressed as fold of change (FOI) in gene expression (2^−ΔΔ*C*t^) occurred in Mimic vs. Scramble groups. ELISA data are expressed as ABS values at 450 nm or in terms of FOI compared to hMSCs transfected with mimic and scramble group. No differences were found between mimic and scramble groups or between experimental time for HIF-1α nuclear levels. Student’s *t* test: *, *p* < 0.05: **, *p* < 0.005 between experimental time.

Concerning the understanding of the LIPUS stimulation effects on hypoxia signaling activation, the dose-dependent reduction in HIF1AN (mRNA), one target of miR-31-5p (*p* = 0.045, Figure 4A), was observed. In addition, miR-31-5p transfection was able to induce the up-regulation of HIF-1α (mRNA) (*p* = 0.003, Figure 4B) and the increase of its target VEGF in terms of mRNA (*p* = 0.008, Figure 4C) and protein (*p* = 0.029, Figure 4D) expression.

### 2.4. The Presence of the miR-31-5p Is Useful to Induce LIPUS Effects on hMSCs

**Figure 5:**
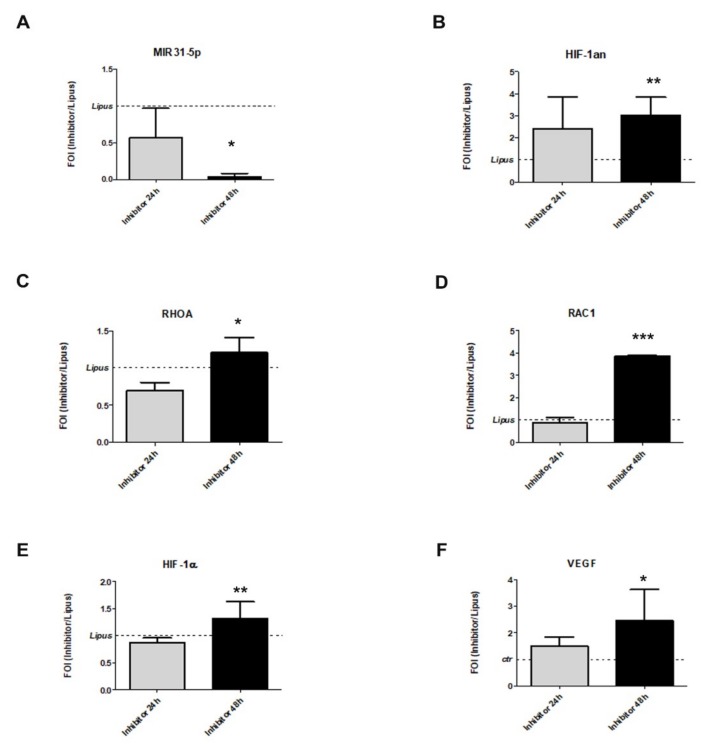
Evaluation if miR-31-5p is useful to induce LIPUS effects on hMSCs in terms of expression of miR-31-5p (**A**), HIF-1AN (**B**), RhoA (**C**), RAC1 (**D**), HIF-1α (**E**), and VEGF (**F**). Quantitative RT-PCR data are expressed as fold of change (FOI) in gene expression (2^−ΔΔ*C*t^) and occurred in inhibitor/LIPUS respect to scramble/LIPUS. Student’s *t* test: *, *p* < 0.05: **, *p* < 0.005; ***, *p* < 0.0005.

With the aim to investigate the role of miR-31-5p in cell responses to LIPUS stimulation, we down-regulated its levels after LIPUS treatments by transfecting cell lines with a specific inhibitor (antimiR-31-5p) and relative scramble (*p* = 0.039, Figure 5A). The inhibition of miR-31-5p in hMSCs treated with LIPUS was related to an increase of miR31-5p targets, such as HIF1an (*p* = 0.007, Figure 5B), RHOA (*p* = 0.010), and RAC1 (*p* < 0.0005) gene expression (Figure 5C,D). Concerning the effects of miR-31-5p inhibition after LIPUS stimulation, we evaluated the modulation of HIF-1α signaling by qRT-PCR and western blot analysis. We noted that the co-treatment (LIPUS/inhibitor) can interfere partially with HIF-1α in terms of mRNA stabilization, as showed by a slight increase of HIF-1αmRNA (*p* = 0.002, Figure 5E) and its target gene VEGF (*p* = 0.016, Figure 5F).

**Figure 6:**
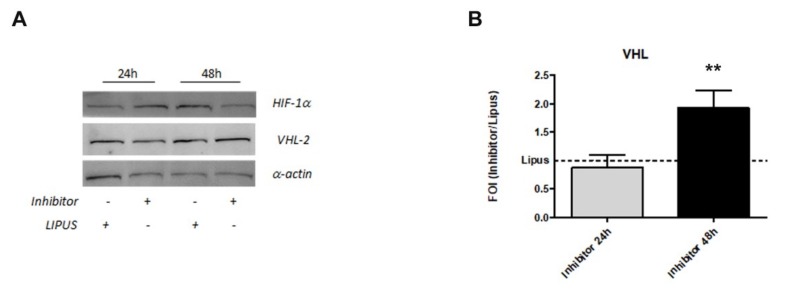
Effects of miR-31-5p induced by LIPUS on HIF-1α protein modulation evaluated by HIF-1α proteins analysis (**A**) and by VHL (**B**) gene expression. Western blot analysis for: HIF1-α and VHL-2, and α-actin proteins were performed on total cells extract (**A**). Quantitative RT-PCR data are expressed as fold of change (FOI) in gene expression (2^−ΔΔ*C*t^) and occurred in Inhibitor/LIPUS compared to Scramble/LIPUS. Student’s *t* test: **, *p* < 0.005.

Moreover, the co-treatment induced a modulation of HIF-1α protein, as demonstrated by western blot analysis of HIF-1α (Figure 6A). In order to evaluate the involvement of LIPUS treatment on HIF-1α protein degradation pathway, we preliminarily investigated the modulation of Von Hippel-Lindau (VHL-protein that induced polyubiquitination and proteosomal degradation of HIF-1α protein) gene and protein after miR-31-5p inhibition and LIPUS stimulation for 24 and 48 h. hMSCs showed an up-regulation of VHL protein (Figure 6A) and VHL mRNA expression (*p* = 0.005) compared to hMSCs stimulated only with LIPUS (*p* = 0.005, Figure 6B).

### 2.5. The Role of miR-31-5p on hMSCs Osteoblast Differentiation

**Figure 7:**
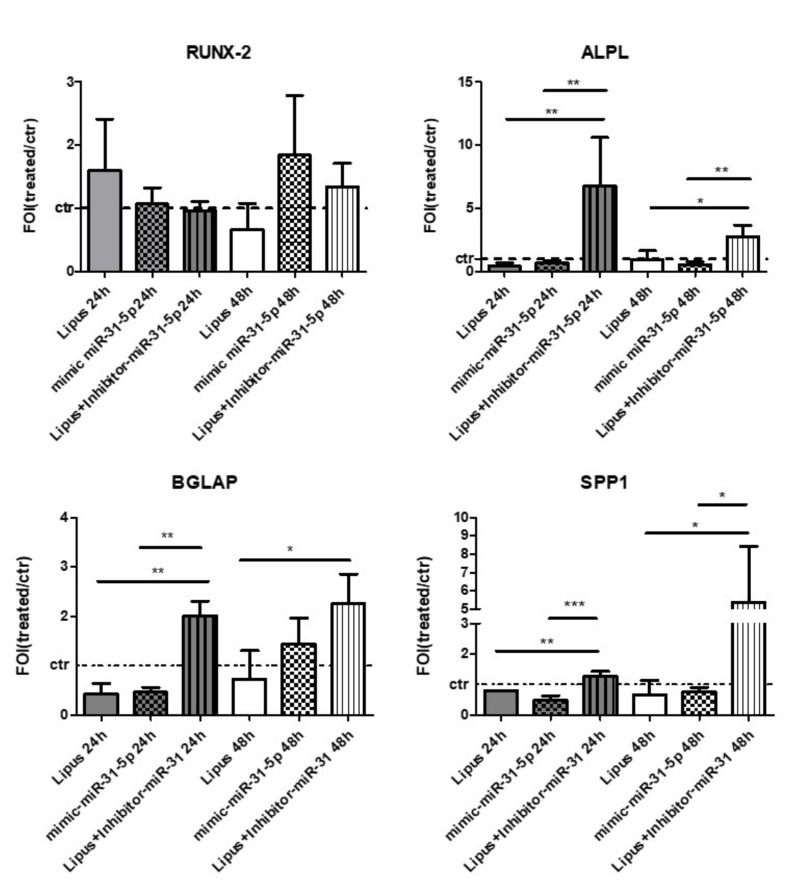
Analysis of osteoblast markers expression, RUNX-2 and ALPL; BGLAP and SPP1, after LIPUS stimulation or miR-31-5p mimic or miR-31-5p inhibitor or negative scramble expression after 24h or 48 h of treatment mean ± SD. Quantitative RT-PCR data are expressed as fold of change (FOI) in gene expression (2^−ΔΔ*C*t^) and occurred in treated groups respect to untreated group. Tukey HSD post hoc comparison test: *, *p* < 0.05; **, *p* < 0.005; ***, *p* < 0.0005.

After the investigation of the miR-31-5p effects on HIF-1α and cytoskeletal signaling modulation, we evaluated the involvement of miR-31-5p on hMSCs osteoblast differentiation. In Figure 7, we report the osteoblast gene expression data (RUNX-2, ALPL, BGLAP, and SPP1) obtained by qRT-PCR analysis of: (i) hMSCs treated for 24 h or 48 h with LIPUS; (ii) hMSCs transfected with mimic miR-31-5p for 24 and 48 h, and (iii) hMSCs transfected with miR-31-5p inhibitor and stimulated by LIPUS for 24 and 48 h. The LIPUS and mimic-miR-31-5p treatments induced similar effects on early osteoblast marker ALPL (*p* < 0.005; *p* < 0.05), while the hMSCs transfected with Inhibitor-miR-31-5p and stimulated by LIPUS improved the expression of late osteoblast markers such as BGLAP and SPP1 (*p* < 0.0005; *p* < 0.005; *p* < 0.05). In addition, miR-31-5p overexpression or inhibition has no effects on SP7 gene expression (Figure S2), confirming the data reported by Baglio et al. [21] and McCully et al. [28]. Finally, starting from the evidence demonstrating that Rho and Rac play a role in the chondrocyte differentiation process, we investigated the involvement of miR-31-5p on this process by evaluating some of the chondrocyte markers using the qRT-PCR approach. However, no modification on chondrocyte markers were observed after treatments. Moreover, Figure S3 reports the modulation of one of the chondrocytes markers, SOX9, despite no modulation after all treatments were observed.

## 3. Discussion

LIPUS treatment induces an acoustic pressure leading to mechanical downstream effects that are translated into a biochemical response, which promotes alterations in gene expression [3,5,29]. In bone tissue, LIPUS-induced mechanotrasduction, is able to regulate bone regeneration and MSCs maintenance.

However, the cross-talk of multiple types of molecules in the regulatory network of the osteogenic differentiation process induced by LIPUS stimulation is not well-defined. Regarding this aspect, we focused our interests on different experimental data that confirm miRNAs as having multi-dimensional roles in the induction of MSCs into osteoblasts, which act at all stages of osteoblast differentiation by inhibiting the negative regulators of signaling pathways operating in these cells or by modulating the signaling pathways involved in this process [16]. The present study identified miR-31-5p as a LIPUS-mechanosensitive miRNA that regulates the hypoxia signaling and cytoskeletal organization in hMSCs in an in vitro model.

We recently reported proteomic data that showed for the first time, the innovative role of LIPUS as regulator of hMSCs stemness and osteoblast differentiation, through the modification of several proteins. In our model, LIPUS stimulation induced, for example, the increase of autophagosomes protein synthesis and mitochondrial proteins [3], a typical signaling involved in hMSCs maintenance, while negatively regulating signaling networks associated to the osteoblast differentiation of hMSCs, such the RhoA/ROCK pathway [3]. RhoA signaling is investigated more as mediator of the hMSCs osteoblast differentiation process; nevertheless, several studies focalized their attention on its cytoskeletal reorganization during hypoxia conditions [7,8,10,12,14]. Hypoxia is detected by HIF-1, -2, and -3 that are intrinsically involved in angiogenesis and osteogenesis during bone development and healing, triggering the processes of direct and indirect ossification. Hypoxia determines many necessary adaptative changes in cells to guarantee their survival, which alter gene expression controlled by HIF-1α. One of these changes is the cytoskeletal rearrangements of cells that allows them to adapt their shape, motility and polarity, division, and the maintenance of multicellular organization [30]. Cross-talk between the hypoxia/HIF-1α and Rho pathways has already been investigated in different cell models, such as cancer cells, fibroblasts, endothelial cells, and MSCs; however, the effects of hypoxia on RhoA levels and activation show many differences among cell types. For example, Gilkes et al. demonstrated in breast cancer cell lines that hypoxia induces an increase of RhoA mRNA and protein, while Xue et al. showed that, in hepatocarcinoma cell lines, hypoxia induced no change in RhoA mRNA expression [31,32]. Regarding hMSCs models, there are many conflicted data concerning the influence of hypoxia on RhoA activity. Vertelov et al. reported that hMSCs enhanced RhoA activation under hypoxia conditions, whereas Raheja et al. showed that hypoxia decreased RhoA activation. Nevertheless, many in vitro studies have suggested that RhoA activation during hypoxia is rapid and time course regulated [14,33].

The present study investigated for the first time the possible involvement of miRNAs in LIPUS stimulation on hypoxia and RhoA signaling regulation, driven by recent evidence about the role of miR-675-5p as inducer of hMSCs osteoblast commitment, triggering HIF-1α and β-catenin signaling [15]. Starting from this observation, through bioinformatic analysis we focused our attention on miR-31-5p, which is reported to regulate the expression of HIF1AN, RhoA and RAC1 in different tumor cell lines [13,28,34,35] and hMSCs [16]. In addition, the role of miR-31-5p on hMSCs differentiation was investigated more by in vitro and in vivo studies. It was found that miR-31-5p suppressed the late differentiation osteogenic stage, acting on Transcription factor Sp7 (SP7) master osteoblast transcription factor, Osteocalcin (BGLAP), and Secreted phosphoprotein 1 (SPP1) protein expression, without affecting Runt-related transcription factor 2 (RUNX2) protein levels, and suggesting that miR-31 specifically influences downstream targets of RUNX2 (mRNA) and thus osteogenesis [21,28].

On the contrary, in vitro evidence suggested that miR-31 regulates Stabilin-2 (STAB2) mRNA, a pivotal regulator of multiple osteogenic-specific genes involved in osteoblast and bone development. The overexpression of miR-31 in MSCs was found to repress STAB2 protein levels and reduce the expression of the osteogenic transcription factors SPP1, Mothers Against Decapentaplegic Homolog 1 (SMAD1), SP7, and BGLAP mRNAs, which may contribute to the maintenance of MSCs in an undifferentiated state [24,36]. In analyzing miR-31 effects on osteogenesis of hMSCs by transfecting exogenous plasmids expressing miR-31 or miR-31 inhibitors, Xie et al. demonstrated that miR-31 negatively regulated the osteogenesis of hMSCs by targeting directly STAB2 (mRNA) [20].

Current data highlights that the ability of LIPUS stimulation to improve hMSCs stemness maintenance and osteoblast hMSCs commitments, is also mediated by the modulation of miR-31-5p expression and, consequently, the regulation of its targets: HIF1AN, RhoA, and RAC1 mRNAs. In particular, the effects of miR-31-5p on HIF1AN (mRNA) induced the activation of HIF1-α signaling, as showed by mRNA expression and nuclear translocation (Figure 2B).

To confirm the role of miR-31-5p in hypoxia and Rho pathways regulation, miR-31-5p in hMSCs cells was overexpressed. After mimic transfection, hMSCs were able to express high levels of HIF-1α and its target gene VEGF, while down-regulating HIF1AN, RhoA, and RAC1 mRNAs. However, when hMSCs cells were co-treated with LIPUS and miR-31-5p inhibitor, hMSCs were able to revert the phenotype displayed after only LIPUS stimulation. In fact, hMSCs showed a down-regulation of miR-31-5p expression levels that is normally induced by LIPUS treatments, while HIF1AN, RhoA, and RAC1 mRNAs were up-regulated. Considering these results, with the hypothetical involvement of miR-31-5p on HIF-1α signaling, the role of VHL pathway on HIF-1α protein regulation was taken into account.

The oxygen-dependent regulation of HIF-1α pathway involves a series of post-translational modifications. The pathway involving VHL, VHL-dependent pathway, regulates HIF-1α stabilization, while that not involving VHL, VHL-independent pathway, regulates HIF-1α transactivation.

In the first mechanism, under normoxia, HIF-1α proteins were found to be good substrates for the action of a group of enzymes called: (i) Prolyl-4- hydroxylases (PHDs) or HIF-1 prolyl hydroxylases (HPH), which induced the hydroxylation of two proline residues; and (ii) arrest-defective-1 (ARD-1), an acetyl transferase enzyme, which induced the acetylation of lysine. Consequently, modified HIF-1α subunits with hydroxylated and acetylated moieties are preferably recognized by VHL and are tagged for ubiquitination and proteasomal degradation of HIF-1α. While VHL-independent pathway is another level of posttranslational modifications of HIF-1α transactivation domain, it does not involve the VHL protein [19,37]. The transcriptional activation of HIF-1α target genes is initiated through the cooperative binding of C-TAD in the HIF-1α and the co-activator CBP/p300; in normoxia, oxygen-dependent hydroxylation of HIF-1α asparagine residue by factor inhibiting HIF-1 (HIF-1AN), also known as asparaginyl hydroxylase, blocks the interaction between the two domains, abrogating the subsequent HIF-1α mediated gene transcription [38].

These preliminary data suggested that the co-treatments LIPUS/miR-31-5p inhibitor was able to up-regulate VHL mRNA and protein expression and interfere with HIF-1α transcription modulating HIF1AN (mRNA), suggesting a possible double role of miR31-5p on HIF-1α signaling regulation modulating VHL-dependent pathway and VHL-independent pathway [39].

For the first time, these data suggest that physical stimulation of LIPUS on hMSCs induces biological downstream effects on HIF-1α and RhoA signal modulations, drives miR-31-5p expression and relative target genes, and determines the maintenance of the balance between undifferentiated and differentiated cells [3], as demonstrated by the evaluation of early and late osteoblast markers expression (Figure 7).

Regarding the role of miR-31-5p on osteoblast commitments of hMSCs, our data revealed that miR-31-5p overexpression or inhibition has no effects on SP7 gene expression (Figure S3), confirming the data previously reported by Baglio et al. and McCully et al. [21,28]. In addition, LIPUS and mimic-miR-31-5p treatments induced similar effects on early osteoblast markers expression, such as RUNX-2 and ALPL, while Lipus+Inhibitor-miR-31-5p improved the expression of late osteoblast markers, such as BGLAP and SPP1, providing the role of miR-31-5p as modulator of osteoblast during the differentiation process (Figure 7).

In conclusion, current data allow us to hypothesize that miR-31-5p is one of the molecular mechanisms through which LIPUS stimulation acts on hMSCs, particularly during the osteoblast commitment of cells. These preliminary data might represent a basis to develop new innovative clinical approaches to bone regeneration therapy, permitting the acceleration of osteoblast regeneration after bone disease or lesion. To confirm our preliminary data, further in vitro investigations will be carried out as well as an in vivo study on hypoxia mouse model [40], in order to understand the in vivo cross-talk between miR-31-5p, hypoxia, and cytoskeletal reorganization with or without LIPUS treatments.

## 4. Materials and Methods

### 4.1. Cell Culture and Reagents

Commercially available hMSCs (Lonza, Walkersville, MD, USA) were cultured in Mesenchymal Stem Cell Growth Medium (MSCGM™ Bullet Kit, Lonza, Walkersville, MD, USA) and maintained at 37 °C in 5% of CO_2_. The cells were split at 70–80% of confluence using StemPro Accutase (Gibco by Life Technologies Italia, Monza, Italy) following the manufacturer’s instructions, and the medium was changed after three days of cultures. Cells were used at an early passage for all experiments.

### 4.2. LIPUS Treatment

The LIPUS exposure device was manufactured by IGEA S.p.A. (Carpi, Italy) [3,5] and consisted on an array of 5 transducers with the ability to produce a signal of 200 μs burst of 1.5 MHz sine waves, repeating at 1 kHz and delivering 30 mW/cm^2^ SATA intensity, transmitted through the bottom of the culture dish via the coupling gel between the ultrasonic transducer and the dish. A calibrated force balance measured the power of the collimated ultrasound beam emitted from the transducer (Ultrasound Power Meters UPM-DT-1AV, Ohmic Instruments, St. Charles, MI, USA) with the mediated power of 33.7 mW/cm^2^. Twenty-four hours before the LIPUS treatment, hMSCs cells were seeded in 6-well plates at the concentration of 150,000 cells/well and all experiments were performed at different time points: 24 and 48 h of treatment. For each experimental time point, hMSCs cultures were divided into two groups according to LIPUS treatment: (a) Untreated group (ctr), cells were cultured and not exposed to LIPUS treatment; and (b) LIPUS group, cells were cultured and treated with LIPUS. Culture plates followed the exposed LIPUS mode as described in Costa et al. [3].

### 4.3. Cell Transfection

For cell transfection, Attractene Transfection Reagent (cat. number 1051531, Qiagen Srl, Milan, Italy) was used following the manufacturer’s indication. Briefly, hMSCs seeded at 150,000 cells/cm^2^ were transfected for 24 and 48 h [15] with 15 pmoles/mL hsa-miR-31-5p mimic (4464066-MC11465, Life Technologies), hsa-miR-31-5p inhibitor (4464084-MH11465, Life Technologies), has-miR-31-3p mimic (4464066-MC12887, Life Technologies) or scrambled negative control (4464058, Life Technologies). In particular, hMSCs transfected with inhibitor and relative scramble were stimulated with LIPUS for 24 and 48 h. For all experimental groups, medium was collected at each experimental time and cells processed for the following assays.

### 4.4. hMSC Viability (WST-1 Test)

The assay is based on the ability of live cells to transform the substrate into formazan, which can be quantified spectrophotometrically by reading at 450 nm (Bio-Rad Microplate Reader-Bio-Rad Laboratories, Hercules, CA, USA). Briefly, the protocol states that WST-1 (stable tetrazolium salt, a colorimetric reagent produced by Roche Diagnostics GmbH, Manheim, Germany) is added to the cell culture at 10% *v*/*v* and that the reading is performed after the appropriate time for the cell line used (4 h). The result is expressed as percentage of viable cells compared to the untreated group.

### 4.5. RNA Extraction and Real-Time PCR

Total RNA was extracted using the commercially available illustraRNAspin Mini Isolation Kit (GE Healthcare, Milan, Italy), according to the manufacturer’s instructions. RNA was reverse transcribed to cDNA using the High Capacity cDNA Reverse Transcription Kit (Applied Biosystems, ThermoFisher Scientific, Rodano, Italy). Quantitative RT-PCR (qRT-PCR) analysis was performed in duplicates for each data point, using custom made primers (Invitrogen, Life Technologies Italy and Qiagen, Monza, Italy) reported in Table 1 and Table 2. The mean threshold cycle was used for the calculation of relative expression using the Livak method against ACTB as the reference gene [41]. For miRNA expression, 250 ng of RNA was reverse transcribed according to the manufacturer’s instructions (cat. number 4366596, TaqMan MicroRNA Reverse Transcription, Applied Biosystems, ThermoFisher Scientific). Taqman probes were used to analyze miR-31-5p (4427975-ID002279, Applied Biosystem, ThermoFisher Scientific), miR-31-3p (4427975- ID002113, Applied Biosystem, ThermoFisher Scientific), and U6 (4427975 Applied Biosystem, ThermoFisher Scientific). Changes in the target miRNA content was calculated in relation to the housekeeping RNU6-1 “RNA, U6 small nuclear 1”.

### 4.6. ELISA Assay

Protein release was measured in the culture medium for VEGF using VEGF Human ELISA Kit (Novex^®^ Cat #KHG011, Life Technologies), according to the manufacturer’s instructions. Data are expressed as fold of change (FOI) of protein release relative to the untreated group or scramble groups.

### 4.7. TransAM Kit

HIF-1α transcriptional factor activity was quantify by an ELISA-based kit (47096, TransAM Kit, Vinci-Biochem, Firenze, Italy) following the manufacturer’s instructions. Briefly, 8 µg of nuclear extracts obtained by using the Nuclear Extract Kit (40010, Vinci-Biochem) were loaded on the coated plate and analyzed by reading at 450 nm with Gen5 Microplate Collection & Analysis Software Data (BioTek Instruments, Inc.^®^, Winooski, VT, USA). The data were expressed as the ratio between HIF-1α protein content and total nuclear extracts (absorbance) or in terms of FOI compared to positive control.

### 4.8. Western Blot Analysis

SDS-PAGE and western blot (WB) were performed according to standard protocols. Briefly, after respective transfection and LIPUS treatments, hMCSs cells were lysed in lysis buffer containing 15 mM Tris/HCl pH 7.5, 120 mM NaCl, 25 mM KCl, 1 mM EDTA, 0.5% Triton X100, and a Halt Protease Inhibitor Single-Use cocktail (100×, ThermoFisher Scientific, Rodano, Italy). Whole lysate (15 µg per lane) was separated using 4–12% NovexBis-Tris SDS-acrylamide gels (Invitrogen, Life Technologies), electro-transferred on nitrocellulose membranes (Bio-Rad Laboratories Srl, Segrate, Milan, Italy), and immunoblotted with the appropriate antibodies. Antibodies against the following proteins were used: HIF-1α (anti-rabbit HIF-1α, Merck Millipore SpA, Vimodrone, Milan, Italy), RhoA (NB100-91273, NovusBiological, Milan, Italy), VHL-2 (VHL (FL-181): sc-5575, Santa Cruz Biotechnology, INC., Heidelberg, Germany), and α-Actin (monoclonal anti-α-actin (1A), sc32251, Santa Cruz Biotechnology, Inc.). The secondary antibodies used for immunoblotting were obtained from ThermoFisher Scientific (ThermoFisher Scientific, Rodano, Italy) and signals were detected using a CCD high-resolution and high-sensitivity detection technology (ChemiDoc™ XRS+ System, Bio-Rad Laboratories Srl, Segrate, Milan, Italy).

### 4.9. Statistical Analysis

Statistical analysis was performed by using R v.3.4.3 software [42]. The Shapiro-Wilk test and Levene test were used to verify normal distribution and homogeneity of variance of data, respectively. Then, Student’s *t* tests were used to compare data. One-way ANOVA followed by the Tukey HSD post hoc comparisons test were used to analyse osteogenic gene expression data within each experimental time frame. Results are reported as mean ± SD at a significant level of *p* < 0.05.

### 4.10. Data Availability

The datasets generated and analyzed during the current study are available in the https://figshare.com/s/ad26012eafe070e11c3a.

## Figures and Tables

**Table 1:** List of gene primers used to study gene expression profiling.

Gene	Primer Forward	Primer Reverse
*HIF-1A* “Hypoxia-inducible factor 1-alpha”	TGATTGCATCTCCATCTCCTACC	GACTCAAAGCGACAGATAACACG
*HIF-1AN* “Hypoxia-inducible factor 1-alpha inhibitor”	TGGGGGCAGCTTACCTCTAA	TGGGTAGAGGCACTCGAAC
*RAC-1* “Ras-related C3 botulinum toxin substrate 1”	TGAAAGCCTTCAGTCCCGTG	TGGTGATGCAGGCTGAACAAT
*RHOA* “Transforming protein RhoA”	GAAAACCGGTGAATCTGCGC	AGAACACATCTGTTTGCGGA
*VEGF* “Vascular endothelial growth factor”	CGAGGGCCTGGAGTGTGT	CGCATAATCTGCATGGTGATG
*VHL* “Von Hippel-Lindau disease tumor suppressor”	GACGGACAGCCTATTTTTGCC	TCCCATCCGTTGATGTGCAA
*SOX9* “Transcription factor SOX-9”	GACTTCTGAACGAGAGCGAGA	CGTTCTTCACCGACTTCCTC
**Reference Gene**		
*ACTB* “Beta-actin”	ATCAAGATCATTGCTCCTCCTGA	CTGCTTGCTGATCCACATCTG

**Table 2:** Qiagen gene primers specific for osteogenic differentiation or involved in the differentiating process. Their expression was normalized to the b-actin housekeeping gene (Table 1).

Gene	Qiagen Primers	Catalog Number
*RUNX2*	Hs_RUNX2_1_SG-QuantiTect Primer Assay	QT00020517
*ALPL*	Hs_ALPL_1_SG-QuantiTect Primer Assay	QT00012957
*BGLAP*	Hs_BGLAP_1_SG-QuantiTect Primer Assay	QT00232771
*SPP1*	Hs_SPP1_1_SG-QuantiTect Primer Assay	QT01008798
*SP7*	Hs-SP7_1_SG-QuantiTect Primer Assay	QT00213514

## Supplementary Materials

Supplementary materials can be found at https://www.mdpi.com/1422-0067/20/7/1569/s1.
