# Supplementary material for: miR-31-5p Is a LIPUS-Mechanosensitive MicroRNA that Targets HIF-1α Signaling and Cytoskeletal Proteins
Source: Int J Mol Sci. 2019 Mar 28;20(7):1569. doi: 10.3390/ijms20071569 (PMC6480017; doi:10.3390/ijms20071569)
Supplement: Supplementary file 1 [file ijms-20-01569-s001.pdf]

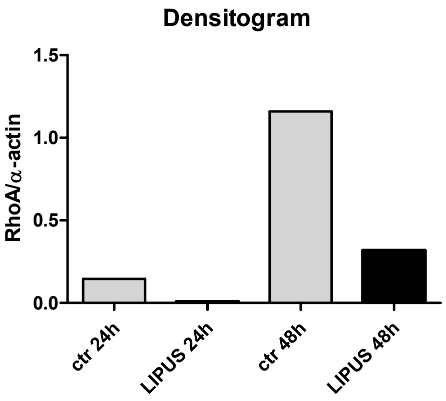

Figure S1. Densitogram of RHOA protein expression compared to α-Actin protein on hMSCs stimulated by LIPUS for 24 h and 48 h and relative control cells.

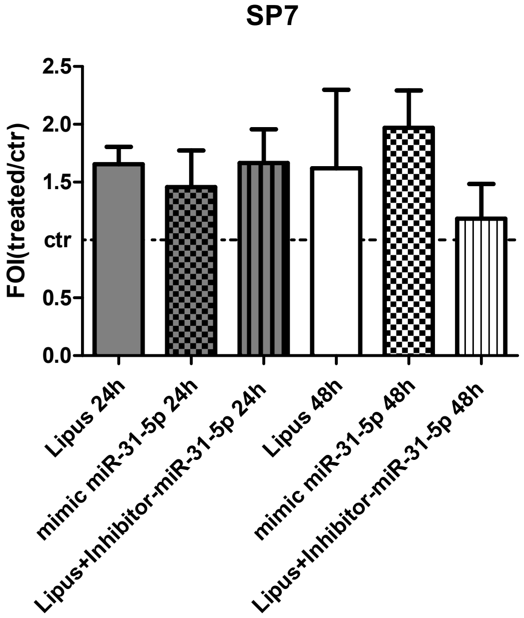

Figure S2. Analysis of osteoblast markers expression SP7, after LIPUS stimulation or miR-31-5p mimic or miR-31-5p inhibitor or negative scramble expression after at 24 or 48 h of treatments. Quantitative RT-PCR data are expressed as fold of change (FOI) in gene expression ($2^{-\Delta\Delta Ct}$) occurred in treated groups vs control group. Data reported were analysed by ANOVA Test and represented as Mean ± SD. Tukey HSD post hoc comparison test: *, $p < 0.05$; **, $p < 0.005$; *** $p < 0.0005$.

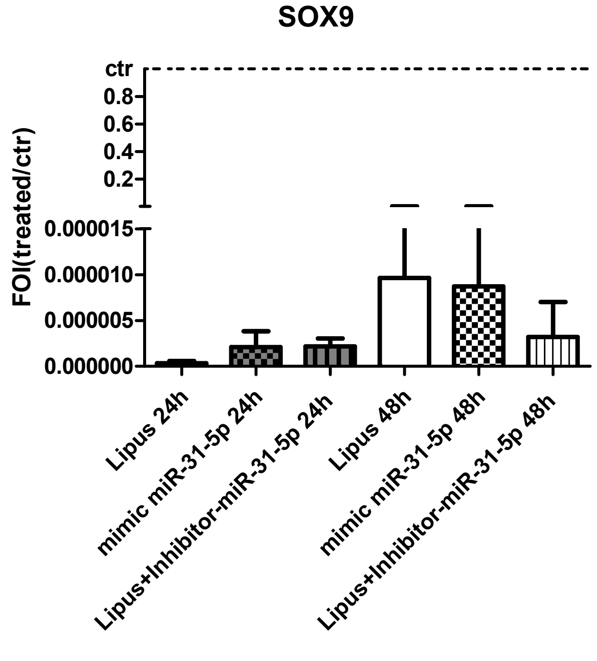

Figure S3. Analysis of SOX9 a chondroblast marker, by Quantitative RT-PCR on samples derived by LIPUS stimulation or miR-31-5p mimic or miR-31-5p inhibitor or negative scramble expression after at 24 or 48 h of treatments. Quantitative RT-PCR data are expressed as fold of change (FOI) in gene expression ($2^{-\Delta\Delta Ct}$) occurred in treated groups vs control group. Data reported were analysed by ANOVA Test and represented as Mean ± SD. Tukey HSD post hoc comparison test: *, $p < 0.05$; **, $p < 0.005$; *** $p < 0.0005$.
